# The grasper-integrated disposable flexible cystoscope is comparable to the reusable, flexible cystoscope for the detection of bladder cancer

**DOI:** 10.1038/s41598-020-70424-0

**Published:** 2020-08-10

**Authors:** Raouf M. Seyam, Omar M. Zeitouni, Tarek M. Alsibai, Abdulrahman J. AlAyoub, Osamah M. Al-Qassab, Mhd A. AlDeiry, Ahmad O. Zino, Hasan S. Hulwi, Alaa A. Mokhtar, Mahmoud Shahbaz, Noor N. Junejo, Mohamed F. Alotaibi, Hassan M. Alzahrani, Khaled I. Alothman, Sultan S. Alkhateeb, Turki O. Al-Hussain, Waleed M. Altaweel

**Affiliations:** 1 Department of Urology, King Faisal Specialist Hospital and Research Centre, Riyadh, Saudi Arabia; 2 College of Medicine, Alfaisal University, Riyadh, Saudi Arabia; 3 Department of Pathology, King Faisal Specialist Hospital and Research Centre, Riyadh, Saudi Arabia

**Keywords:** Bladder, Bladder cancer

## Abstract

Flexible cystoscopy under local anaesthesia is standard for the surveillance of bladder cancer. Frequently, several reusable cystoscopes fail to reprocess. With the new grasper incorporated single-use cystoscope for retrieval of ureteric stents, we explored the feasibility of using it off-label for diagnosis and the detection of bladder cancer. Consecutive diagnostic flexible cystoscopies between Mar 2016 and Nov 2018 were reviewed comparing the reusable versus the disposable cystoscopes. A total of 390 patients underwent 1211 cystoscopies. Median age was 61.5 years (SD 14.2, 18.8–91.4), males 331 (84.9%) and females 59 (15.1%). Indication for cystoscopy was prior malignancy in 1183 procedures (97.7%), haematuria 19 (1.6%) or bladder mass 7 (0.6%). There were 608 reusable and 603 disposable cystoscopies. There was no significant difference between groups at baseline in age, sex, BMI, smoking status, or prior tumor risk category. There was no significant difference in positive findings (123/608, 20.2% vs 111/603, 18.4%, *p* = 0.425) or cancer detection rates (95/608, 15.6% vs 88/603, 14.4%, *p* 0.574) among the two groups, respectively. We conclude that the disposable grasper integrated cystoscope is comparable to reusable cystoscope in the detection of bladder cancer.

## Introduction

The number of new bladder cancer cases worldwide in 2018 was 549,000, accounting for 200,000 deaths the same year^1^. Bladder cancer ranked the 10th most common cancer worldwide, excluding skin cancer and the second most common cancer of the genitourinary tract after prostate cancer. Bladder cancer is common in men with an age-standardized risk of 9.6, which is four times more common than in women of 2.4. Non-muscle invasive bladder cancer (NMIBC) has a high rate of recurrence and needs long-term vigilant management, making it the costliest cancer treated per patient^2^. Surveillance and progression of the disease contribute to the high cost^3^. Cystoscopy is the cornerstone of the evaluation and follow-up of NMIBC^4^. Non-adherence to cystoscopy surveillance results in doubling of the risk of tumor progression^5^. Adjunctive biopsy or resection and urine cytology provide the histopathological diagnosis upon which definitive management depends. Imaging^6,7^, and urinary marker tests^8,9^ are complementary, and none has yet become an acceptable alternative to cystoscopy^10^. Flexible cystoscopy under local anaesthesia is associated with better patient acceptance, particularly in men^11,12^.

In our hospital, the urology department performs an average of 70–80 diagnostic flexible cystoscopies per month. Most of these procedures are for surveillance of NMIBC. The protocol for surveillance is following the American Urological Association guidelines^10^. In our service, around 15–20 flexible scopes are available at one time, and each scope undergoes numerous sterilization and utilization cycles. The standard of reprocessing of flexible scopes in our institution is sterilization using low heat hydrogen peroxide gas as recommended by the manufacturer. Applying a robust cystoscope reprocessing cycle, lead to the failure of several scopes to pass the leakage test, resulting in interruption of service, and delay of patient appointments. A new grasper incorporated disposable flexible cystoscope (GDC) has been clinically tested for the removal of ureteric stents^13^. A recent multicentre European study concluded that the new scope has a good image quality, deflection mechanism, and manoeuvrability^14^. We elected to explore the feasibility of its use off-label for diagnostic purposes after introducing the new GDC in our department for retrieval of ureteric stents.

Our aim in this study is to evaluate whether the use of GDC is comparable to reusable scopes in the detection of bladder cancer.

## Methods

**Figure 1:**
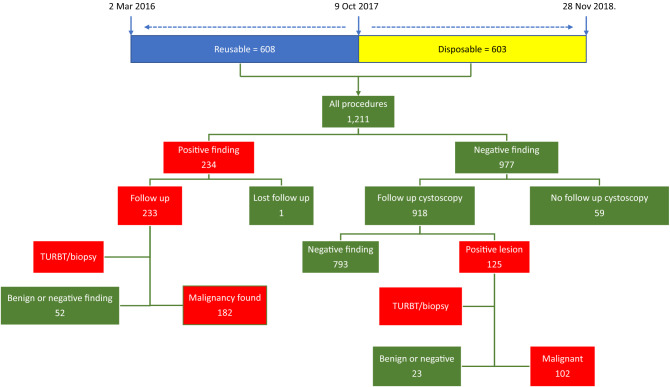
Flowchart of the cystoscopy procedures.

We retrospectively reviewed the electronic charts of patients that underwent flexible cystoscopy under local anaesthesia consecutively between 2 Mar 2016 and 28 Nov 2018. Exclusively reusable scopes were used from 2 Mar 2016 to 4 Oct 2017 and exclusive disposable scopes from 9 Oct 2017 to 28 Nov 2018 (Fig. 1). The time frame for the inclusion of procedures was 581 days for the reusable and 415 days for the disposable scopes. The King Faisal Specialist Hospital and Research Centre Clinical Research Committee and Ethics Committee approved the project on 5 Feb 2018. All methods were performed following the guidelines and regulations (Research Advisory Council project number 2181020). The Ethics Committee has waved the informed consent because of the retrospective study design. From the date of the approval onwards, new patients were added to the database according to the routine management protocol indications with no changes because of the study. The indication of cystoscopy was bladder tumor surveillance or suspicion of bladder cancer. Exclusion criteria were paediatric patients under age 18 years, an indication for evaluation of lower urinary tract symptoms, stent removal, rigid cystoscopy, or cystoscopy under general or regional anaesthesia. The procedures were performed twice weekly by four urologists throughout the study (RS, AM, MS, and NJ). A single day surgery operating room with standard staffing, sterilization, and draping was adopted in all cases. We compared two cystoscopy systems, the reusable, flexible scopes (Storz, Germany) and the GDC (Isiris, Coloplast, Denmark). We evaluated age, sex, height, weight, smoking status, previous tumor pathology, prior tumor risk stratification, positive cystoscopy findings of a tumor or a suspicious lesion, and subsequent resection or biopsy pathology result. Risk stratification of each NMIBC was according to pathological staging and grading, as described elsewhere^14^. Prior T2–T3 bladder tumors that underwent bladder preservation protocol and upper urothelial tract T3 high-grade tumors post nephroureterectomy were categorized among the high-risk tumors.

### Statistics

We started the disposable cystoscopy service on 9 Oct 2017. We included at least 600 consecutive patient-cystoscopy procedures before and after that date for reusable and disposable cystoscopies, respectively. Each procedure was considered an individual entry toward the final analysis.

We analysed data using descriptive statistics, *t*-test for continuous variables (2-tailed), and for categorical variables, Fisher exact tests (1-sided) for 2 × 2 tables or Pearson chi-square tests (2-sided) for 2 × 3 or larger tables. Logistic regression was used to detect confounders impacting the result of cystoscopy. Testing equality of two mean counts was used to analyse the number of repeated cystoscopies per patient per group. Significance was assumed when *p* ≤ 0.05). We used SPSS software for statistical analysis (IBM SPSS Statistics for Windows, Version 25.0. Armonk, NY: IBM Corp).

## Results

A total of 390 patients underwent 1211 consecutive cystoscopy procedures between Mar 2016 and Nov 2018. The patient cystoscopy procedures were 608 for the reusable cystoscopy and 603 for the disposable cystoscopy. Indication for cystoscopy was surveillance for prior malignancy in 1183 procedures (97.7%), while 28 (2.6%) procedures were for patients who had symptoms suggestive of malignancy namely haematuria (19, 1.6%), bladder mass on imaging (7, 0.6%) or unexplained chronic cystitis (2, 0.2%).

The baseline characteristics of unique patients are shown in Table 1. No comparison between unique patients was possible because each patient underwent both types of cystoscopies at different time points (Supplementary Table S1). Furthermore, several patients had therapeutic procedures during follow up like transurethral resection of bladder tumor (TURBT), some had recurrences, and some had a change of grade or stage of the pathology. We analysed the procedure frequency for each patient and within each group (Table 2). There was no statistical difference in the frequency of repeated cystoscopy per patient among the reusable and disposable cystoscopy procedures. We analysed the stage, grade, and risk stratification for patients before each procedure (Table 3). No prior tumor pathology was available for cystoscopies carried out for haematuria, a bladder mass, or symptoms suggestive of malignancy. Absent previous pathology was excluded from the analysis of baseline tumor stage, grade, and risk category. A negative cystoscopy finding, and an unknown smoking status were included as separate variables in the study. The prior pathology of cystoscopy surveillance for T2–T3 bladder tumors, which underwent bladder preservation protocol (3.9%) and post UTUC nephroureterectomy (1.4%) were included in the analysis.

**Table 1:** Baseline characteristics of unique patients (n = 390).

	Median (SD)	Range
Age (years)	61.5 (14.2)	18.8–91.4
Weight (kg)	79.4 (16.7)	40.0–143.3
Height (meter)	1.7 (0.1)	1.4–1.9

	Frequency	Percent
Sex		
Female	59	15.1
Male	331	84.9
Smoking		
Ex-smoker	12	3.1
Non-smoker	143	36.7
Smoker	151	38.7
Unknown	84	21.5
Prior pathology		
Not available^a^	23	5.9
CIS	27	6.9
T1	59	15.1
T2–T3	17	4.4
Ta	258	66.2
UTUC	6	1.5
Prior tumor grade^b^		
High	153	41.7
Low	214	58.3
Risk stratification^b^		
High	174	47.4
Intermediate	12	3.3
Low	181	49.3
Type of cystocopy		
Reusable	311	79.7
Disposable	332	85.1

*UTUC* upper tract urothelial carcinoma.

^a^Indication of cystoscopy was haematuria or mass lesion on imaging.

^b^n = 367 after exclusion of absent prior tumor pathology.

**Table 2:** Frequency of cystoscopy sessions per patient.

Number of cystoscopies per patient	Reusable cystoscopy frequency	Disposable cystoscopy frequency
Once	132	144
Twice	98	124
Three times	56	46
Four times	13	17
Five times	11	1
Six times	1	
Mean number of cystoscopies	1.945	1.81
Variance	1.09	0.77
*p*		0.925

**Table 3:** Baseline characteristics of patients per procedure.

	All cystoscopies		Reusable cystoscopy		Disposable cystoscopy		*p*
	n	Mean (SD)	n	Mean (SD)	n	Mean (SD)	
Age (years)	1211	60.7 (13.8)	608	60.4 (13.9)	603	60.9 (13.8)	0.855
Weight (kg)	1211	81.0 (16.7)	608	80.6 (16.8)	603	81.4 (16.5)	0.526
Height (meter)	1211	1.66 (0.08)	608	1.7 (0.1)	603	1.7 (0.1)	0.887

	n (%)		n (%)		n (%)		
Sex							
Female	138 (11.4)		63 (10.4)		75 (12.4)		0.278
Male	1073 (88.6)		545 (89.6)		528 (87.6)		
Smoking status							
Non-smoker	426 (35.2)		222 (36.5)		204 (33.8)		0.363
Ex-smoker	42 (3.5)		25 (4.1)		17 (2.8)		
Smoker	480 (39.6)		237 (39)		243 (40.3)		
Unknown	263 (21.7)		124 (20.4)		139 (23.1)		
Tumor stage							
No prior pathology^a^	28 (2.3)		9 (1.5)		19 (3.2)		
Ta	832 (70.3)		424 (70.8)		408 (69.9)		0.391
CIS^b^	94 (7.9)		54 (9)		40 (6.8)		
T1	193 (16.3)		94 (15.7)		99 (17)		
T2–T3	47 (4)		19 (3.2)		28 (4.8)		
UTUC^c^	17 (1.4)		8 (1.3)		9 (1.5)		
Total	1183		599		584		
Tumor grade^a^							
High grade	537 (45.4)		267 (44.6)		270 (46.2)		0.599
Low grade	646 (54.6)		332 (55.4)		314 (53.8)		
Total	1183		599		584		
Tumor risk category^a^							
Low risk	527 (44.5)		269 (44.9)		258 (44.2)		0.268
Intermediate risk	44 (3.7)		17 (2.8)		27 (4.6)		
High risk	612 (51.7)		313 (52.3)		299 (51.2)		

*CIS* carcinoma in situ, *SD* standard deviation.

^a^Patients with no prior pathology were excluded (n = 28); indication for cystoscopy was haematuria or a bladder mass on imaging.

^b^Includes any stage with CIS.

^c^UTUC: upper tract urothelial carcinoma, TaLG one case, TaHG two cases, and T3HG 14 cases.

There was no difference between the groups for underlying risk factors. A regression analysis was conducted to identify factors that impacted the result of cystoscopy towards having a positive finding (Table 4). The type of cystoscopy did not have a significant impact on the outcome. The significant factors affecting the positive result were gender, being a current smoker, and belonging to intermediate or high-risk stratification. Of note is that the prior stage alone did not affect the detection rate, and the preceding pathology with high grade negatively correlated with the positive result. These factors, in combination and with the frequency of tumor recurrence, were ingredients of the risk category, which was associated with a significant increase with a factor of 0.78–1.87 for a positive result finding.

**Table 4:** Logistic regression for factors leading to a positive finding in cystoscopy.

	B	S.E	Sig	Exp(B)	95% C.I. for EXP(B)	
					Lower	Upper
Cystoscopy type	− 0.230	0.156	0.140	0.795	0.586	1.078
Gender	0.603	0.296	**0.042**	1.828	1.023	3.266
Age	0.002	0.006	0.720	1.002	0.990	1.015
Smoking status						
Ex-smoker	0.679	0.451	0.132	1.972	0.815	4.774
Non-smoker	0.377	0.230	0.100	1.459	0.930	2.287
Smoker	0.595	0.238	**0.012**	1.813	1.138	2.889
Prior pathology						
CIS	0.954	1.076	0.375	2.597	0.315	21.379
T1	0.392	1.067	0.713	1.480	0.183	11.974
T2–T3	0.937	1.116	0.401	2.552	0.286	22.738
Ta	1.215	1.048	0.247	3.370	0.432	26.308
High grade	− 0.669	0.316	**0.034**	0.512	0.276	0.951
Prior risk stratification						
High risk	0.783	0.304	**0.010**	2.189	1.206	3.974
Intermediate risk	1.873	0.337	**0.000**	6.511	3.361	12.611

B values for the logistic regression equation; C.I. Confidence interval; CIS carcinoma in situ; Exp(B) odds ratios; S.E. Standard error of the mean.

Positive cystoscopy lesions were subjected to resection or biopsy (Fig. 1). The positive cystoscopy finding was 19.3% for all procedures, 20.2% for reusable cystoscopy and 18.4% for disposable cystoscopy (Table 5). The cancer detection rates were 15% for all procedures, 95/608, 15.6% for reusable and 88/603, 14.4%for disposable cystoscopies. A comparison between the reusable and disposable cystoscopy groups showed no significant difference in the rate of positive findings or cancer detection.

**Table 5:** Cystoscopy findings.

	All cystoscopies	Reusable cystoscopy	Disposable cystoscopy	*p*
	n (%)	n (%)	n (%)	
Number of cystoscopy procedures	1211	608	603	
Cystoscopy findings				
Negative cystoscopy finding	977 (80.7)	485 (79.8)	492 (81.6)	0.425
Positive cystoscopy finding	234 (19.3)	123 (20.2)	111 (18.4)	
Post cystoscopy pathology				
Benign or negative pathology^a^	51 (21.9)	28 (22.8)	23 (20.9)	0.989
CIS, T2–3	15 (6.4)	8 (6.5)	7 (6.4)	
T1	21 (9)	11 (8.9)	10 (9.1)	
Ta	146 (62.7)	76(61.8)	70 (63.3)	
Malignant total	182 (78.1)	95 (77.2)	87 (79.1)	0.753^b^
Total	233 (100)	123(100)	110 (100)	
Negative cystoscopy or benign	1029 (85)	513 (84.4)	516 (85.6)	0.574
Malignancy	182 (15)	95 (15.6)	87 (14.4)	
Grade^b^				
High grade	69 (37.9)	36 (37.9)	33 (37.9)	0.559
Low grade	113 (62.1)	59 (62.1)	54 (62.1)	

^a^One case was lost for follow up.

^b^Malignant compared to benign or negative pathology.

Cystoscopies, which had a negative finding were further followed up for the subsequent procedure to confirm the negative result (Fig. 1). Of the 977 procedures who had negative cystoscopy results 59 had no follow up cystoscopy and were considered lost for follow up and excluded from the analysis. After a mean of follow up of 217.7, SD 117.9 days, 86.4% of the 918 negative procedures remained negative, whereas 13.6% converted to positive (Table 6). There was no statistical difference between the two groups. The cancer detection rate showed a significant association between the higher risk category at baseline and a higher stage and grade for the final pathology (Supplementary Table S2).

**Table 6:** Follow up of cystoscopies with negative findings.

	Reusable		Disposable		Total		p
	Mean	SD	Mean	SD	Mean	SD	
Follow up duration (days)	215.8	125.3	219.6	110.2	217.7	117.9	0.157

	n	%	n	%	n	%	p
Confirmed negative	394	85.7%	399	87.1%	793	86.4%	0.564
Became positive	66	14.3%	59	12.9%	125	13.6%	
Total	460		458		918		

All procedures were done without a prophylactic antibiotic with strict operative room sterilization protocol. None of the patients required analgesia following the procedure. The complications following cystoscopy procedures were rare and not included in the analysis. One procedure was complicated by urosepsis, and another by haematuria in the reusable scope group. Both patients required hospitalization and active treatment. Difficulties encountered during cystoscopy using the disposable scope were limited flexion and image resolution. The grasper incorporated scope has limitations of the degree of angular deflection due to the presence of the forceps in the distal portion (Supplementary Video S1). A retrograde J manoeuvre was adopted to visualize the anterior bladder wall, and examination of the ureteric orifices was deferred to the end of the procedure when the bladder was full (Supplementary Video S2). The J manoeuvre adequately visualized small lesions at the bladder neck (Supplementary Video S3). There was no quantification of the urologist’s perception of the quality of the image. There was an agreement among the four urologists conducting the procedures that the disposable scope image had a lower resolution than the reusable scope. The difference in resolution did not affect the detection of small lesions, as seen in comparison with rigid cystoscopy and high definition images during subsequent TURBT or biopsy (Supplementary Video S4).

## Discussion

White light flexible cystoscopy under local anaesthesia remains the principal method for diagnosis and surveillance of bladder cancer. Investigators tried to improve the cancer detection rate by applying different technologies as a modification or replacement of white light cystoscopy^15^. Some though superior to white light cystoscopy, have not gained full acceptance in daily practice because of complexity, while others still need to validate their role in NMIBC management.

There are several challenges to achieve high-level disinfection or sterilization of flexible endoscopes^16^. The recommendation for cystoscope processing is either high-level disinfection or sterilization^17^. Channelled flexible scopes require a complex process to ensure adequate high-level disinfection or sterilization^18,19^. Inadequate processing, residual contaminants, or scope damage add to the difficulty of achieving effective disinfection^20^. Even when the manufacturer recommendations and guidelines for disinfection were followed, outbreaks of infection transmitted through endoscopes occurred^21^. The standard of practice in our hospital is to sterilize flexible cystoscopes to ensure patient safety. The protocol of sterilization demands rigorous washing, testing of equipment for leakage or damage, proper transportation, adequate sterilization time, post sterilization quality indicators, and proper storage. Unfortunately, the life span of the scope becomes shorter with diligent maintenance of sterilization. When some of the scopes fail, the remaining are overused. Such an occurrence spirals quickly into multiple scopes failing. In 2017 such a crisis occurred, leading to disruption of our busy cystoscopy tumor surveillance service. It is not known what exactly the factor is contributing to the high failure rate. Frequent reprocessing and sterilization of each scope might have contributed. This failure rate was not observed in other hospitals where high-level disinfection was the standard of reprocessing flexible scopes.

At the same time, we introduced the GDC in our service and was readily available over the shelf. The introduction of a single-use flexible cystoscope for J-stent removal met the expectation of simplifying the procedure logistics and moving it from the OR to the office. These scopes were associated with cost reduction, less stent dwell time, less morbidity, and improved patient satisfaction^22^. A previous study compared the physical characteristic in the lab for the grasper integrated scope versus five other cystoscope brands^14^. The grasper integrated scope was the second-best in image quality but had the narrowest field of vision. A factor that might have affected the good image quality in the study is that the tested grasper incorporated scope was new while the other scopes were previously used. With no grasper in the working channel, all the other scopes had a better irrigation flow rate and a better deflection range.

We used these scopes off label to detect bladder tumors. Our preliminary findings gave the impression that the disposable scopes were comparable to the reusable scopes. A pilot study showed that the GDC was useful in visualizing all parts of the urinary bladder and had a cancer detection rate of 14%. The urology department discussed the study, and there was a consensus agreement to use the GDC for diagnostic purposes in the evaluation and surveillance of NMIBC. After Oct 2017, all diagnostic cystoscopies were exclusively using the GDC. Reusable scopes were available as a second-line method if the urologist performing the procedure is uncomfortable in visualizing the whole bladder mucosa. No session required a backup reusable scope examination. As the grasper incorporated scope has its limitations and was not designed with the aim of diagnosis, scepticism rose whether it is comparable to the standard reusable scope in detecting bladder tumors. The forceps at the tip of the scope significantly limits its ability to bend in a retrograde fashion. Other caveats included a lack of a working channel for biopsy or fulguration. Despite these disadvantages, the mere presence of an off-shelf cystoscope dominated our cystoscopy. Adapting certain manoeuvres lead to overcoming these difficulties. A retrograde J manoeuvre was adopted to visualize the anterior bladder wall, and examination of the ureteric orifices was deferred to the end of the procedure when the bladder was full. At the time of our study, no approved disposable cystoscope was available in our country. Sheath cystoscopes were developed to overcome the chemical disinfection hazard and to increase cystoscope availability in a busy urology service^23–25^. Although these scopes have been around for more than a decade, they are not available to our hospital.

Our department intended to evaluate in a retrospective study the cancer detection rate and modify our recommendations accordingly.

### Bias and study design

The study cystoscopy sessions were consecutively performed; there was no selection of the type of cystoscopy. The only factor determining which scope used was the cut-off date of the 9th Oct 2017. As a retrospective study, several inherent biases may confound the results. The analysis of the baseline characteristics of each patient per procedure did not show a significant difference in any of the risk factors (Table 3). Furthermore, logistic regression did not show that the type of cystoscopy had an impact on the kind of result (Table 4). Admittedly, a prospective randomized trial in patients powered for detection of 14% cancer rate is more informative. The difficulty anticipated is that at least 600 patients are needed for a none-inferiority study in each arm^26,27^. The number of cystoscopies for 1200 patients will reach thousands, and the period for recruitment quite long. Alternatively, a trial where the disposable scope is compared to the reusable scope as a gold standard will provide answers on sensitivity, specificity, and positive and negative rates. Such a study design requires repeating a procedure in the same patient at the same time for the sake of research, which is not justifiable and does not parallel similar studies where a non-invasive diagnostic tool is tested against a gold standard.

The pathology of the prior tumor may affect the detection rate as non-papillary tumors, or smaller solitary lesions may be challenging to detect. On the other hand, high-grade tumors with positive cytology may persuade the examiner to perform a more thorough cystoscopy examination. This phenomenon is evident in one study where the cancer detection rate was enhanced by prior knowledge of urine markers^28^. In our study, however, the distribution of the previous pathology, including tumor stage, grade, and risk category, was not different among the groups (Table 1). A detection rate for a suspicious lesion was higher for the reusable compared to the disposable groups (20.2% vs 18.2%), but the difference was non-significant (Table 5). Similarly, the cancer detection rate was higher for the reusable scope (15.6% vs 14.4%); however, reusable scopes detected more benign or false lesion compared to the disposable scopes (22.8% vs 20.9%).

### Confirmation of results

There is a concern that cystoscopies with negative results may have missed the diagnosis. Follow up cystoscopy, however, confirmed the negative result in 793/918 (86.4%) cystoscopies with a mean follow up of at least 3 months (Fig. 1, Table 6). All cystoscopies that had a change of diagnosis at follow up (125/918, 13.6%) were subjected to biopsy or TURBT. A total of 102/918 (11.1%) procedures showed malignancy in the definitive pathology. Considering the long follow up lapse of a mean 218 days, it is not known whether these lesions were missed out or were new recurrences. However, in a multicentre European trial, NMIBC was characterized by a high recurrence rate ranging from 15 to 61% in 1 year^29^. This tumor behaviour may indicate that the new lesions in our study were recurrences rather than missed lesions. Besides, the comparison of result data among the cystoscopy type groups did not show a significant difference in the rate of new lesions (Table 6)”.

Cytology was not used to confirm the negative findings of cystoscopy. Any pre-cystoscopy cytology showing malignant or atypical cells even in the absence of a visible lesion mandated upper tract imaging and a random bladder biopsy. The result of the biopsy rather than the cytology was taken as an indicator of whether the cystoscopy missed out the diagnosis.

### Limitations

A shortcoming of our study is that it is not designed to compare a new test against a gold standard test in the same patient. Therefore, sensitivity, specificity, false-positive or negative values are not calculated, and verification bias is not estimated. Our study is retrospective and lacks randomization. Cystoscopy procedures were in two consecutive periods, not in a parallel fashion. Potential problems are different baseline data leading to bias and variation in the urologists performing the procedures. However, in our study, we showed that there were no significant differences among groups at baseline, and logistic regression did not show an impact of cystoscopy type on results. Furthermore, the procedures were performed by the same four urologists, each with experience with flexible cystoscopy surveillance at least for 5 years. Another limitation of the study is the lack of objective assessment of patient comfort, acceptance, and pain. The extra pressure exerted may cause more discomfort to the patient than the conventional reusable scope as the GDC needs special manoeuvres to see all blind areas of the urinary bladder. Only two grade 3 complications in the reusable cystoscopy procedure occurred, and these were not included in the analysis. A shortcoming is that the study did not include an assessment of minor complications (grades 1–2).

The study involves multiple cystoscopy sessions in the same patient. We considered each session a separate entry. The example patient in Supplementary Table S1 illustrates the numerous procedures, underlying pathology changes, multiple treatments, and different results in a single patient. It is not possible to categorize such a patient in one group. The authors, therefore, decided to assess procedures rather than patients. The drawback is that the same patient is repeatedly reported. This repetition is more prominent in the sex, height, and weight categories. Duplication also occurred when the prior pathology continues to be the same for several consecutive cystoscopy session. However, as the data for each session is unique regarding prior pathology, cystoscopy findings and postresection pathology, we believe that the cancer detection rate is valid.

In the current budget oriented medical service, the cost is an important variable. In this study, however, we aimed to establish similarity in cancer detection rate as the primary factor for the continuation of using the GDC for bladder cancer diagnosis. Therefore, our current report study lacks a cost analysis. Insight into cost-effectiveness is in favor of the GDC. Cost-effective analysis for JJ stent removal was reported in favor of the disposable scope when factoring in the operative room versus office-based procedure cost^30^. In our study, both types of cystoscopies were carried in the same place with similar staffing and OR protocol. The only difference was the scope cost and cost of reusable scope sterilization.

We conclude that the development of a dedicated diagnostic disposable cystoscope has several advantages but has not materialized in everyday use. The disposable grasper integrated cystoscope is comparable to a reusable cystoscope in the detection of bladder cancer. The grasper disposable scope may fill the gap as an alternative to the conventional reusable cystoscope.

## Supplementary information

Supplementary Information.

Supplementary Video 1

Supplementary Video 2

Supplementary Video 3

Supplementary Video 4

## Data Availability

All data analysed during this study are included in the Supplementary Information file (Anonymous patient data file.xlsx).
