# Supplementary material for: The grasper-integrated disposable flexible cystoscope is comparable to the reusable, flexible cystoscope for the detection of bladder cancer
Source: Sci Rep. 2020 Aug 10;10:13495. doi: 10.1038/s41598-020-70424-0 (PMC7417573; doi:10.1038/s41598-020-70424-0)
Supplement: Supplementary file 1 — Supplementary Information. [file 41598_2020_70424_MOESM1_ESM.pdf]

Title:
The grasper-integrated disposable flexible cystoscope is comparable to the reusable, flexible cystoscope for the detection of bladder cancer.

Authors:
Raouf M. Seyam[1*], Omar M. Zeitouni[2], Tarek M. Alsibai[2] Abdulrahman J. AlAyoub[2], Osamah M. Al-Qassab[2], Mhd A. AlDeiry[2], Ahmad O. Zino[2], Hasan S. Hulwi[2], Alaa A. Mokhtar[1], Mahmoud Shahbaz[1], Noor N. Junejo[1], Mohamed F. Alotaibi[1], Hassan M. Alzahrani[1], Khaled I. Alothman[1], Sultan S. Alkhateeb[1], Turki O. Al-Hussain[3], and Waleed M. Altaweel[1].
[1]Department of Urology, King Faisal Specialist Hospital and Research Centre, Riyadh, Saudi Arabia, [2]College of Medicine, Alfaisal University, Riyadh, Saudi Arabia and [3]Department of Pathology, King Faisal Specialist Hospital and Research Centre, Riyadh, Saudi Arabia.

Supplementary Table S1. Patient number 1x5xx6. Example of multiple procedures and pathologies for the same patient.

Patient ID	Cystoscopy type	Cystoscopy Date	Previous pathology	Previous Pathology date	Risk Category	Cystoscopy Finding	Cystoscopy Finding Pathology
1x5xx6	Reusable	14-Nov-16	TaLG	11-Oct-15	Low	Negative	
1x5xx6	Reusable	15-May-17	TaLG	11-Oct-15	Low	Positive	TaHG
1x5xx6	Reusable	30-Aug-17	TaHG	8-Jun-17	High	Negative	
1x5xx6	Disposable	27-Nov-17	TaHG	8-Jun-17	High	Negative	
1x5xx6	Disposable	26-Feb-18	TaHG	8-Jun-17	High	Negative	
1x5xx6	Disposable	14-May-18	TaHG	8-Jun-17	High	Negative	
1x5xx6	Disposable	12-Nov-18	TaHG	8-Jun-17	High	Positive	TaHG

Supplementary Table S2. The relationship between baseline risk category and the postoperative pathology.

	Baseline risk category*			
	Low or intermediate risk** N (%)	High risk N (%)	Total N (%)	p
Post resection stage				
CIS, T1-3	4 (4.0)	28 (37.8)	32 (18.4)	0.000
Ta	95 (96.0)	46 (62.2)	141 (81.5)	
Total	99 (100)	74 (100)	173 (100)	
Post resection grade				
High grade	20 (20.2)	43 (58.1)	63 (36.4)	0.000
Low grade	79 (79.8)	31 (41.8)	110 (63.6)	
Total	99 (100)	74 (100)	173 (100)	

Supplementary Video files:

1. Supplementary Video S1. The range of flexion of the reusable versus the disposable cystoscopes.
2. Supplementary Video S2. The J manoeuvre and visualization of the ureteric orifices.
3. Supplementary Video S3. The J manoeuvre adequately visualizes small tumors at the bladder neck.
4. Supplementary Video S4. Comparison between disposable cystoscope image and rigid cystoscopy with a high definition camera in the same patients.

Supplementary Data file:

Rebuttal SPSS data set ID coded No MRN for NSR.xlsx
